# Evaluation of Turkish Ophthalmologists' Opinions on Surgical Waste During Cataract Surgery

**DOI:** 10.1155/joph/9972323

**Published:** 2025-05-11

**Authors:** Bahadır Utlu, Betül Dertsiz Kozan, Yunus Emre Erat

**Affiliations:** ^1^Ophthalmology, Erzurum Faculty of Medicine, University of Health Sciences, Erzurum, Türkiye; ^2^Ophthalmology, Health Sciences University, Diyarbakir Gazi Yaşargil Health Applıcation and Research Center, Diyarbakir, Türkiye

**Keywords:** carbon footprint, cataract surgery, disposable materials, surgical waste

## Abstract

**Purpose:** Objective: We aimed to investigate the attitudes of ophthalmologists toward the excessive amount of waste generated during modern cataract surgery practices.

**Methods:** Survey study and a prospective, cross-sectional, descriptive study. An online questionnaire consisting of 23 questions was directed to ophthalmologists practicing in Erzurum Province and its surroundings. Duplicate responses were prevented by allowing only one submission to the survey. Participants were asked to complete the questionnaire only if they performed cataract surgery.

**Results:** The study included 102 ophthalmologists through an online survey. Approximately 43% of the participants performed between 200–500 cataract surgeries per year. 90% of the respondents believed that operating room waste was excessive and should be reduced. Among the drivers of waste/garbage generation in the study, the most common response (78%) was the widely recognized safety benefits of disposables. Again, when asked about the main drivers for using disposable instruments in ophthalmic surgery, the most common response was reduced liability (77%). 80% of respondents said that device and supply manufacturers should consider the environmental/carbon footprint in product design. When asked to what extent it reduces your willingness to use a material or medication in more than one patient, the highest preferred answer was the risk of endophthalmitis, while the least preferred answer was decreased efficiency.

**Conclusion:** In conclusion, most ophthalmologists agree that a lot of waste is generated during surgery and that more widespread use of sterilizable materials rather than disposables could reduce waste.

## 1. Introduction

Sustainability is a term defined by the United Nations in the late 20th century to describe the measures to be taken against the dangers such as climate change, the decrease of the green environment and forests, and the extinction of biodiversity, which emerged as a result of the increasing environmental damage caused by the speed of production and consumption, industrial load, and increasing urbanization in the world. Irregular, nonlinear anthropogenic developments such as the increase in world temperature, climate changes, and the increase of infectious diseases can cause irreversible and self-reinforcing changes in the ecosystem with a continuous vicious cycle. This situation has recently been called a “climate crisis” by major powers, including developed countries, and within this framework, the “Kyoto Protocol”, which entered into force in 2009 under the leadership of the UN, was signed. According to this protocol, the aim is for countries that accept this protocol to reduce their carbon emissions and keep them balanced at levels that will not create a dangerous effect on the climate [1].

The health system is on both sides of the definition of the climate crisis. While the treatment burden of the population suffering from the consequences of the negative effects of the global climate is a burden on the health system, on the other hand, the increase in the number of examinations, obesity, the disproportionate development of surgical options due to esthetic concerns, and defensive medicine, which has emerged as a result of laws that increase the number of unnecessary examinations and treatments, have become a significant driver of climate change. Although this situation is difficult to calculate, the results are not surprising. According to one report, the health system accounts for approximately 5% of the carbon emissions in a given country [2].

Due to the large elderly population and the high volume of major surgical operations, ophthalmology seems likely to be one of the largest contributors to the carbon emissions of the health system [3]. Cataract surgery is the most common elective eye surgery procedure and also requires a large amount of sterile, single-use surgical instruments, energy, and consumables to avoid surgical complications such as endophthalmitis, which can lead to blindness [4]. As a result, the materials, energy consumption, and hospital infrastructure required to perform the surgery contribute to significant carbon emissions [5]. The carbon footprint of a single cataract operation in the UK has been estimated to be 181.8 kg CO_2_e (equivalents), the majority of which is from single-use materials [6, 7]. Although it is highly effective in restoring vision, there is increasing concern about its environmental impact, particularly in terms of CO_2_ emissions [6]. In this case, although it is clear that healthcare professionals can contribute to the adoption of more environmentally friendly practices with their awareness of sustainability, we aimed to investigate the awareness of ophthalmologists against this increasing situation in our study.

## 2. Methods

The study was designed as a prospective, cross-sectional, descriptive study. A 22-question survey developed by Chang [8] and previously used in North America and Europe was made available online using Google Forms (insert citation) and directed to ophthalmologists performing cataract surgery in and around Erzurum. Only one submission was allowed to the survey to prevent duplicate responses. Participants were asked to complete the survey only if they had undergone cataract surgery. The online survey was sent to all members of the Turkish Ophthalmology Association Erzurum Trabzon Branch. Only 102 responses were received from approximately 250 physicians. In Table 1, the responses to the question “How would you rate the practical impact of each of the following factors in increasing surgical waste during cataract surgery?” are presented.  Table 2 contains the responses to the question “What do you think are the main factors that necessitate the use of single-use surgical materials/instruments in cataract surgery?” Table 3 presents the responses to the question “To what extent do you agree or disagree with the following statements?” Table 4 shows the responses to the question “Please describe your situation regarding the use of the following drugs and consumables (assuming they are properly cleaned and sterilized) in multiple cataract surgeries.” Table 5 presents the responses to the question “To what extent do the following factors reduce your willingness to reuse a material or medication for multiple patients?” Finally, Table 6 contains the responses to the question “Which of the following can be done by you?” The highest and lowest selected responses were analyzed. Permission was obtained from the Health Sciences University Erzurum Faculty of Medicine Clinical Research Ethics Committee for this study (ethics committee meeting date and number: 12.02.2024/05-03).

The SPSS 20 package program was used for statistics and analysis. Descriptive statistics are presented as frequency and percentage for categorical data and mean and standard deviation for numerical data. The Kolmogorov–Smirnov test was used to examine the normality of numerical variables. One way ANOVA was applied for normally distributed variables, and the Kruskal–Wallis test was applied for non-normally distributed variables. Statistically, *p* < 0.05 was accepted as the significance limit.

## 3. Result

The study included 102 ophthalmologists via an online survey. Fifty-four percent of the participants were female, and the mean age was 42.9 + −0.9 years. Approximately 43% of the participants performed between 200–500 cataract surgeries per year. 30% of the participants had 6–10 years of cataract surgery experience.

Ninety percent of the participants believed that operating room waste was excessive and should be reduced. When asked in Table 1 to select the “highest impact” among the drivers of waste generation, 82% identified “the widely accepted safety benefits of single-use items”, 64% stated that “manufacturers prevent the reuse of single-use surgical materials through usage instructions to limit their liability, even if safety measures are in place”, and 54% indicated that “manufacturers guide surgeons to use single-use materials to increase market share”. The lowest response was given by “patient preference” at 28%.

When participants were asked in Table 2 about the main factors that make the use of single-use surgical materials/instruments mandatory in cataract surgery, the highest percentage response was “reduced legal/medical liability” at 80%. This was followed by “patient medical safety” at 76% and “surgical team safety” at 68%. The lowest response was given by 18% who indicated “patient and family preference”.

In Table 3, when participants were asked “To what extent do you agree with the following?” regarding their agreement with certain statements, 82% of participants stated that “medical device and consumable manufacturers should consider environmental/carbon emissions in their product designs,” 80% believed that “healthcare systems should adopt practices and policies that protect the environment and reduce operating room carbon emissions,” and 78% indicated that “medical device and consumable manufacturers should use recyclable materials in their packaging.”

In Table 4, when participants were asked to explain how they reused certain drugs and materials (assuming they were properly cleaned and sterilized) in multiple cataract surgeries, the highest reuse rates were for mydriatic drops (74%), phaco tips (60%), and *I*/*A* tips (56%). The lowest reuse rate was for sutures at 20%.

In Table 5, when asked “To what extent do the following factors reduce your willingness to reuse a material or medication for multiple patients?,” 92% of participants indicated that “major impact” was caused by the risk of endophthalmitis. When asked about factors that would increase their willingness to reuse material or medication, 60% of participants identified “cost savings.” Similarly, when asked “To what extent do the following factors affect your willingness to reuse sterilized single-use medical materials and devices under the necessary conditions?,” 60% selected “product performance” as the key factor.

In Table 6, when asked “Which of the following applies to you?,” 40% of participants indicated that “the re-sterilization and reuse of single-use surgical instruments obtained from surgeries (if necessary regulations are in place)” was feasible, 38% selected “removal of full-body coverage garments (using only face coverings),” and 38% chose “surgical staff should use the same surgical mask throughout the day.” Additionally, in all tables, the comparison of the answers given by North American and European survey participants and Turkish physicians is shown in tabular format.

## 4. Discussion

According to the results of our study, ophthalmologists are concerned about the climate crisis and are highly motivated to reduce surgical waste. In particular, there was a consensus that disposable supplies can be reused for multiple patients on the same day or if sterilized under appropriate conditions. In the current situation, the awareness of the surgical team on this issue appears to be high, similar to several previous studies using this survey.

In a study conducted using a survey developed by Chang and Thiel, a total of > 1500 cataract surgeons working in North America and Europe were asked, and 93% of the participants reported that they believed that OR waste was excessive and should be reduced. In the same study, the majority of the participants reported that they believed that many consumables should be resterilized or reused while maintaining sterilization, and 90% were concerned about global warming and believed that efforts should be made to reduce the surgical carbon footprint [8]. The results are highly consistent when compared with Turkish ophthalmologists. Given the high level of awareness in this context, perhaps the first change or practice to be made to solve the problem of waste in ophthalmology surgical practice is for all surgeons to decide to work together in a common decision framework.

When the responses of the participants in the survey conducted in North America to some critical points were compared with the responses of the participants in our study, it was observed that there were some differences. According to Turkish ophthalmologists, the main factor that necessitates the use of disposable surgical materials in cataract surgery is the reduction of legal/medical liability. This situation was answered with a high majority among the participants in the survey conducted in North America as the performance of the instruments. Again, in the other question asked, the answer “Healthcare systems should adopt practices and policies that protect the environment and reduce carbon emissions in operating rooms” was accepted with a high majority, while according to the results of the survey conducted in North America, the expectation that the number of disposable materials will increase is high. Again, according to the results of our study, the idea that “Regulatory agencies limit the surgeon's discretion to reuse consumables” is the highest response to the answer “Publicly accepted safety benefits of disposable surgical consumables”. According to the results of our study, it was stated that the same mydriatic drops are used in more than one case in cataract surgery, while the answer to this answer in the results of the survey conducted in North America was IA types [8, 9]. While Turkish physicians stated that they have reduced their desire to use any material or drug on more than one patient due to the risk of endophthalmitis, the more dominant response according to physicians participating in the survey conducted in North America is malpractice liability. While the factor that increases the desire to use a material or drug on more than one patient is savings, according to the results of the survey conducted in North America, it is waste reduction. If the necessary arrangements are made in cataract surgery, Turkish ophthalmologists are willing to resterilize and use single-use instruments, while ophthalmologists participating in the survey conducted in North America answered that they would use the same surgical mask all day at most. In summary, it is clear that regulatory institutions in our country limit the discretionary authority of surgeons to reuse consumables and that there could be serious developments in sustainable ophthalmology if the necessary legal arrangements are made. Although this situation is similar to the situations stated in the studies in the literature, it is clearly seen that medicolegal situations are more dominant, especially in the responses of Turkish ophthalmologists.

One of the most common causes of waste, according to the participants in our study, is the existence of industrial restrictions on the resterilization or reuse of consumables and medical products. Participants also believed that the manufacturer was driven to produce more single-use products due to the potential for increased commercial profit, the shared responsibility for damages, and a lack of concern about carbon emissions. Furthermore, most participants wanted the manufacturer to provide more options for recyclable products and give more decision-making power on which products could be reused and when. Khor et al. reported that excluding recyclable materials, the average waste produced per case was 0.669 kg and emphasized that adhering to the 3 Rs (reduce, reuse, and recycle) principles in waste processing could reduce environmental impact [10]. According to the survey, participants believed that topical and intracameral medications, phacoemulsification tips, irrigation solutions, and tubes, as well as incision knives and cannulas, should be reused.

It is known that the disposable materials required for cataract surgery are a significant source of carbon emissions. It has been estimated that the disposable materials produced for just one phaco surgery cause an average of 2–5 kg of CO_2_ emissions per surgery, even during the sterilization process [10]. Another study has shown that disposable cassettes generate approximately 4 times more plastic waste per 1000 phaco surgeries per day than reusable cassettes [6].

However, there are some obstacles to this. The most important of these is the legal dimension of the situations that may arise after complications due to infections and how to convince physicians against the negative effects on patient morbidity, keeping in the background the principle of “first do no harm,” which is one of the basic principles of the medical profession. In our study, in order to support these statements, it was seen that the most important factor encouraging the use of single-use devices is the risk of endophthalmitis and that medical/legal security is important in decision-making. However, the concerns experienced in this case are not always valid. For example, in the study where daily reusable cassettes were used instead of single-use cassettes, no endophthalmitis was observed during cataract procedures within 2 years [6]. Again, consecutive bilateral cataract surgery is every surgeon's nightmare. However, in the studies conducted, it is accepted that the infection risk of bilateral cataract surgery is now low with the widespread use of intracameral antibiotics [11].

It is no surprise that modern ophthalmology practice generates an incredible amount of waste every day. Advances in surgical practice are increasing the scope of available interventions. Furthermore, as the world population grows and ages, the demand for ophthalmologic surgery is certain to increase. Even in developed countries where cataract surgeries are performed on average 100 per capita per year, demographic changes are expected to increase the number of major ophthalmic surgical cases over the next 20 years. For example, in the United Kingdom, the projected increase is 52% for cataracts, 49% for glaucoma requiring medical treatment, and 64% for age-related macular degeneration. The WHO predicts that the number of elderly people in the world will increase to 40 million by 2025, with a longer life expectancy [12].

Our study has several limitations. The most important of these is the small number of Turkish ophthalmologists who participated in the study. Furthermore, the translation of the previously conducted survey by Chang into the local language seems to reduce the significance of the study. More concrete studies on waste in our clinic are needed.

Finally, ophthalmology practice may negatively impact the climate due to the high volume of surgery and required supplies. Ophthalmologists agree that waste in surgery is excessive and that the use of disposable supplies can be reduced. Furthermore, more research and innovation should be prioritized to address the increasing carbon emissions of surgery.

## Figures and Tables

**Table 1:** “How would you evaluate the practical impact of each of the following as factors that increase surgical waste generation in operating rooms during cataract surgery?” answers to question.

How would you evaluate the impact of each of the following on practice as factors that increase surgical waste generation in operating rooms during cataract surgery? (*n*:102)	High impact (ESCRS) (OICS) (%)		Moderate impact (ESCRS) (OICS) (%)		Little or no impact (ESCRS) (OICS) (%)	
Publicly recognized safety benefits of disposable surgical supplies	80.39	(71) (74)	19.61	(26) (22)	0	(3) (4)
Positive contributions of disposable surgical consumables to surgical performance	56.86	(40) (33)	35.29	(44) (44)	7.84	(15) (24)
Depending on the absolute demand of disposable surgical consumables by the surgical team	41.18	(42) (26)	39.22	(40) (45)	9.8	(18) (28)
Ophthalmologists do not reuse surgical supplies whenever possible	33.33	(41) (33)	41.18	(41) (37)	25.49	(18) (30)
During cataract surgery, the surgical team has to unpack many consumables that they think are necessary for the procedure	43.14	(38) (37)	41.18	(38) (39)	15.69	(24) (24)
Hospital management/infection committee/ministry of health policies limit the surgeon's discretion regarding the reuse of materials even if necessary safety precautions are maintained	37.25	(70) (71)	41.18	(26) (24)	11.76	(24) (5)
Regulatory bodies/ministry of health limit the surgeon's discretion regarding the reuse of his/her materials even if the necessary safety precautions are provided	45.1	(58) (74)	43.14	(36) (21)	11.76	(6) (5)
Mostly because patients want disposable instruments to be used during the operation	27.45	(65) (82)	23.53	(28) (15)	49.02	(7) (3)
To limit their liability, manufacturers prevent the reuse of disposable surgical supplies through instructions for use, even if necessary safety precautions are provided	62.75	(17) (7)	35.29	(23) (19)	1.96	(60) (74)
Manufacturers are directing surgeons/technicians toward the use of disposable surgical supplies, with the belief that it will have a positive impact on their economic indicators	52.94	(67) (70)	41.18	(27) (26)	5.88	(6) (4)
Lack of awareness of environmental/carbon emission harms	43.14	(74) (77)	43.14	(24) (20)	13.73	(1) (3)
Disposable surgical supplies that are unnecessarily packaged multiple times, creating excessive waste	39.22	(73) (65)	47.06	(23) (26)	13.73	(4) (10)

**Table 2:** In your opinion, what are the main factors that make it necessary to use disposable surgical materials/instruments in cataract surgery? answers to the questions.

In your opinion, what are the main factors that make it necessary to use disposable surgical materials/instruments in cataract surgery? (*n*:102)	High impact (ESCRS) (OICS) (%)		Moderate impact (ESCRS) (OICS) (%)		Little or no impact (ESCRS) (OICS) (%)	
Occurrence of performance differences due to reuse of surgical materials	45.10	(47) (38)	45.10	(35) (42)	9.80	(18) (20)
Reducing legal/medical liability	78.43	(50) (66)	21.57	(36) (26)	0.00	(14) (8)
Medical safety of the patient	74.51	(64) (49)	23.53	(25) (40)	1.96	(11) (12)
Safety of the surgical team	66.67	(26) (16)	29.41	(36) (48)	3.92	(38) (36)
Preference of patients or their relatives	17.65	(10) (6)	39.22	(28) (29)	43.14	(61) (65)
Cost savings of hospital management	21.57	(41) (26)	43.14	(34) (36)	35.29	(26) (39)
Reduced personnel handling requirements (e.g. cleaning and sterilization)	33.33	(55) (45)	41.18	(38) (45)	15.69	(8) (10)
Operating room modernization and increased efficiency	45.10	(45) (37)	43.14	(42) (47)	11.76	(13) (16)
Producer profit	39.22	(50) (62)	35.29	(27) (20)	25.49	(23) (18)
Complies with health guidelines	60.78	(48) (65)	29.41	(40) (26)	9.80	(12) (9)

**Table 3:** “To what extent do you agree or disagree with the ideas below?” answers to the question.

To what extent do you agree or disagree with the ideas below? (*n*:102)	Absolutely I agree (ESCRS) (OICS) (%)		I agree (ESCRS) (OICS) (%)		I am undecided (ESCRS) (OICS) (%)		I do not agree (ESCRS) (OICS) (%)		I strongly disagree (ESCRS) (OICS) (%)	
Manufacturers of medical devices and consumables must use recyclable contents in their packaging	76.47	(76) (72)	19.61	(18) (18)	1.96	(4) (7)	1.96	(1) (1)	0	(1) (1)
Medical device and consumable manufacturers should consider environment/carbon emissions in their product designs	80.39	(85) (76)	17.65	(10) (16)	1.96	(3) (5)	0	(0) (1)	0	(1) (1)
Manufacturers of medical devices and consumables must design their products for reuse	72.55	(74) (81)	25.49	(18) (13)	1.96	(5) (5)	0	(1) (1)	0	(1) (0)
Manufacturers of medical devices and consumables should give ophthalmologists more decision-making power in their instructions for use (e.g., single-use may be recommended but reuse may be allowed when necessary conditions are met)	68.63	(60) (75)	25.49	(29) (18)	3.92	(5) (5)	0	(4) (2)	1.96	(1) (1)
Institutions that make health-related laws and guidelines should give ophthalmologists more decision-making authority to reuse materials, drugs, and medical devices used for surgery	66.67	(64) (81)	23.53	(25) (14)	7.84	(6) (3)	0	(4) (1)	1.96	(1) (0)
Health systems should adopt practices and policies that protect the environment and reduce carbon emissions in operating rooms	78.43	(83) (78)	13.73	(12) (14)	5.88	(4) (5)	1.96	(0) (1)	0	(1) (2)
The medical societies to which I belong should advocate for reducing the carbon footprint in operating rooms	74.51	(79) (71)	15.69	(15) (16)	7.84	(4) (7)	1.96	(1) (3)	0	(1) (3)
We need more studies to evaluate the safety of reuse when necessary	70.59	(67) (68)	25.49	(19) (19)	3.92	(10) (7)	0	(2) (3)	0	(2) (2)

**Table 4:** Describe your use of the following pharmaceuticals and supplies (assuming they have been properly cleaned and sterilized) in more than one case in cataract surgery”answers to the question.

Describe your use of the following pharmaceuticals and supplies (assuming they have been properly cleaned and sterilized) in more than one case in cataract surgery. (*n*:102)	I am currently using (ESCRS) (OICS) (%)		I might consider using it (ESCRS) (OICS) (%)		I do not want to use it (ESCRS) (OICS) (%)		I am not sure (ESCRS) (OICS) (%)	
Mydriatic	72.55	(43) (48)	19.61	(48) (51)	5.88	(7) (1)	1.96	(2) (1)
Antibiotics	47.06	(43) (45)	17.65	(43) (53)	31.37	(10) (1)	3.92	(4) (1)
Nsaıds	37.25	(34) (38)	33.33	(52) (59)	25.49	(9) (1)	3.92	(5) (2)
IOP-lowering medications	45.1	(32) (42)	27.45	(55) (55)	21.57	(10) (1)	5.88	(3) (1)
Anesthetic	47.06	(42) (43)	29.41	(48) (55)	19.61	(7) (1)	3.92	(2) (1)
Antibiotics administered intracamerally	41.18	(48) (32)	11.76	(48) (32)	41.18	(39) (63)	5.88	(3) (2)
Alpha-agonists/mydriatics	47.06	(33) (34)	25.49	(33) (34)	23.53	(50) (61)	3.92	(3) (3)
Miotics	41.18	(28) (20)	29.41	(28) (20)	25.49	(53) (73)	3.92	(5) (2)
Lidocaine	39.22	(39) (30)	19.61	(39) (30)	37.25	(45) (65)	3.92	(4) (2)
Capsule paint	39.22	(21) (10)	25.49	(21) (10)	33.33	(53) (80)	1.96	(6) (3)
Corticosteroids (for example, triamcinolone)	37.25	(21) (16)	19.61	(21) (16)	41.18	(55) (76)	1.96	(7) (4)
Commercially packaged solutions	31.37	(19) (11)	21.57	(19) (11)	37.25	(61) (84)	9.8	(7) (2)
Solutions mixed by operating room nurse	31.37	(23) (15)	19.61	(23) (15)	43.14	(47) (67)	5.88	(9) (8)
Types of phacoemulsification	58.82	(48) (38)	13.73	(42) (54)	27.45	(8) (5)	0	(2) (3)
IA types	54.9	(48) (41)	19.61	(40) (49)	25.49	(9) (6)	0	(3) (4)
Tubing sets	52.94	(21) (7)	21.57	(55) (69)	23.53	(17) (17)	1.96	(8) (7)
Irrigation solution/bottle	50.98	(26) (8)	31.37	(47) (70)	17.65	(21) (15)	0	(7) (6)
Capsulotomy needle/cystotome	45.1	(32) (13)	13.73	(33) (59)	39.22	(29) (22)	1.96	(7) (6)
Small diameter cannulas	49.02	(18) (27)	13.73	(38) (47)	31.37	(36) (21)	5.88	(8) (6)
Metal knives	41.18	(18) (14)	27.45	(43) (64)	27.45	(31) (18)	3.92	(8) (4)
Nonmetallic surgical devices (iris and capsule retractors)	45.1	(16) (9)	23.53	(48) (63)	27.45	(27) (20)	3.92	(9) (8)
Sütüres (E.G. other half)	19.61	(12) (3)	23.53	(33) (56)	43.14	(44) (32)	13.73	(11) (9)

**Table 5:** “To what extent do the following factors reduce your desire to use a material or medication on more than one patient?” answers to the question.

**To what extent do the following factors reduce your desire to use a material or medication on more than one patient? (*n*:102)**	**Major impact (ESCRS) (OICS) (%)**		**Minor impact (ESCRS) (OICS) (%)**		**Not effective (ESCRS) (OICS) (%)**	

Risk of endophthalmitis	90.2	(64) (48)	9.8	(26) (38)	—	(9) (15)
TASS risk	84.31	(40) (43)	11.76	(43) (39)	3.92	(17) (18)
Surgeon safety concern	80.39	(18) (11)	15.69	(38) (37)	3.92	(44) (52)
Decreased productivity	47.06	(13) (7)	39.22	(36) (31)	13.73	(50) (62)
Malpractice	78.43	(41) (51)	19.61	(41) (38)	1.96	(18) (11)

**To what extent do the following factors increase your willingness to use a material or medication on more than one patient? (*n*:102)**	**Major impact (ESCRS) (OICS) (%)**		**Minor impact (ESCRS) (OICS) (%)**		**Not effective (ESCRS) (OICS) (%)**	

Saving	58.82	(47) (63)	39.22	(46) (35)	1.96	(7) (2)
Waste reduction	31.37	(76) (78)	56.86	(21) (20)	7.84	(3) (2)
Reduced carbon footprint	35.29	(73) (66)	50.98	(23) (27)	13.73	(4) (7)
Decreased productivity	23.53	(49) (63)	60.78	(37) (33)	15.69	(14) (4)

**To what extent do the following factors affect your desire to use disposable medical supplies and devices that have been resterilized under necessary conditions? (*n*:102)**	**Major impact (ESCRS) (OICS) (%)**		**Minor impact (ESCRS) (OICS) (%)**		**Not effective (ESCRS) (OICS) (%)**	

Cost	58.82	(56) (59)	37.25	(39) (33)	3.92	(6) (8)
Security risk	72.55	(78) (72)	25.49	(18) (22)	1.96	(4) (6)
Product performance	82.35	(77) (79)	17.65	(21) (18)	—	(2) (3)
Relationship, affiliation, and/or trust with the company	37.25	(31) (33)	43.14	(46) (39)	19.61	(22) (27)
Health regulations	56.86	(55) (72)	35.29	(37) (24)	7.84	(8) (5)
Patient perception	47.06	(12) (16)	27.45	(52) (44)	25.49	(35) (39)
Environmental/carbon emission considerations	45.1	(79) (58)	37.25	(18) (30)	17.65	(3) (12)

**Table 6:** Which of the following can be done for you? answers to the question.

Which of the following can be done for you? (*n*:102)	Already done (ESCRS) (OICS) (%)		Willing to consider (ESCRS) (OICS) (%)		Unwilling to take into consideration (ESCRS) (OICS) (%)		I am not sure (ESCRS) (OICS) (%)	
Eliminating full body covering (use face covering only)	37.25	(47) (44)	21.57	(41) (51)	31.37	(10) (4)	9.8	(3) (1)
Do not make the patient wear a hospital gown (the patient stays in his own clothes)	11.76	(50) (56)	23.53	(27) (34)	52.94	(19) (7)	11.76	(4) (3)
Not changing surgical gowns between each case (surgeon and operating room nurse)	15.69	(10) (4)	23.53	(45) (60)	50.98	(38) (28)	9.8	(6) (7)
Not changing surgical gloves between each case	11.76	(3) (1)	21.57	(14) (16)	56.86	(75) (77)	9.8	(8) (7)
Those working in the operating room must use the same surgical mask all day long	37.25	(49) (64)	29.41	(33) (31)	25.49	(13) (4)	7.84	(5) (1)
Resterilization and use of disposable instruments obtained from surgeries (e.g. in the case of necessary regulations)	39.22	(14) (7)	37.25	(70) (84)	15.69	(11) (5)	7.84	(5) (4)
I use short cycle/same day sterilization techniques (flash autoclave cycle)	37.25	(26) (26)	37.25	(57) (65)	17.65	(10) (5)	7.84	(7) (5)
Bilateral cataract surgery	17.65	(14) (8)	17.65	(45) (48)	54.9	(32) (34)	9.8	(10) (10)
I send home the pharmaceuticals used in surgery (e.g. topical antibiotics) to patients coming from the operating room	29.41	(27) (26)	31.37	(55) (67)	29.41	(9) (4)	9.8	(9) (2)
I preserve unused surgical supplies and give them to patients for use	25.49	(20) (26)	25.49	(70) (71)	35.29	(7) (2)	13.73	(3) (1)

## Data Availability

The data supporting this study's findings are available from the corresponding author upon reasonable request.

## References

[1] Watts N., Amann M., Arnell N. (2021). The 2020 Report of the Lancet Countdown on Health and Climate Change: Responding to Converging Crises. *The Lancet*.

[2] Lenzen M., Malik A., Li M. (2020). The Environmental Footprint of Health Care: A Global Assessment. *The Lancet Planetary Health*.

[3] Taboun O. S., Orr S. M., Pereira A., Choudhry N. (2023). Factors Contributing to the Carbon Footprint of Cataract Surgery. *Journal of Cataract & Refractive Surgery*.

[4] Talley N. J., Stanley F., Lucas T., Horton R. (2021). Health and Climate Change MJA-Lancet Countdown Report: Australia Gets Another Failing Grade in 2020 But Shows Signs of Progress. *The Lancet*.

[5] Rizan C., Steinbach I., Nicholson R., Lillywhite R., Reed M., Bhutta M. F. (2020). The Carbon Footprint of Surgical Operations. *Annals of Surgery*.

[6] Kallay O., Sadad R., Zafzafi A., Motulsky E. (2024). Cataract Surgery and Environmental Sustainability: A Comparative Analysis of Single-Use versus Reusable Cassettes in Phacoemulsification. *Bio Medical Journal Open Ophthalmology*.

[7] Thiel C. L., Schehlein E. M., Ravilla T. (2017). Cataract Surgery and Environmental Sustainability: Waste and Lifecycle Assessment of Phacoemulsification at a Private Healthcare Facility. *Journal of Cataract & Refractive Surgery*.

[8] Chang D. F. (2020). Survey of Cataract Surgeons’ and Nurses’ Attitudes toward Operating Room Waste. *Journal of Cataract & Refractive Surgery*.

[9] Chang D. F., Elferink S., Nuijts R. M. M. A. (2023). Survey of ESCRS Members’ Attitudes Toward Operating Room Waste. *Journal of Cataract & Refractive Surgery*.

[10] Khor H. G., Cho I., Lee K. R. C. K., Chieng L. L. (2020). Waste Production From Phacoemulsification Surgery. *Journal of Cataract & Refractive Surgery*.

[11] Morris D. S., Wright T., Somner J. E. A., Connor A. (2013). The Carbon Footprint of Cataract Surgery. *Eye*.

[12] Pascolini D., Mariotti S. P. (2012). Global Estimates of Visual Impairment: 2010. *British Journal of Ophthalmology*.

